# Comparative Analysis of Orbital Morphology Accuracy in 3D Models Based on Cone-Beam and Fan-Beam Computed Tomography Scans for Reconstructive Planning

**DOI:** 10.3390/jcm14155541

**Published:** 2025-08-06

**Authors:** Natalia Bielecka-Kowalska, Bartosz Bielecki-Kowalski, Marcin Kozakiewicz

**Affiliations:** 1 Department of Oral Mucosal and Periodontal Diseases, Medical University of Lodz, 251 Pomorska St., 92-213 Lodz, Poland; natalia.bielecka-kowalska@umed.lodz.pl; 2 Department of Maxillofacial Surgery, Medical University of Lodz, 113 Żeromskiego St., 90-549 Lodz, Poland; marcin.kozakiewicz@umed.lodz.pl

**Keywords:** orbital reconstruction, cone-beam computed tomography, multidetector computed tomography, patient-specific implants, computer-aided design

## Abstract

**Background/Objectives:** Orbital reconstruction remains one of the most demanding procedures in maxillofacial surgery. It requires not only precise anatomical knowledge but also poses multiple intraoperative challenges. Limited surgical visibility—especially in transconjunctival or transcaruncular approaches—demands exceptional precision from the surgeon. At the same time, the complex anatomical structure of the orbit, its rich vascularization and innervation, and the risk of severe postoperative complications—such as diplopia, sensory deficits, impaired ocular mobility, or in the most serious cases, post-traumatic blindness due to nerve injury or orbital compartment syndrome—necessitate the highest level of surgical accuracy. In this context, patient-specific implants (PSIs), commonly fabricated from zirconium oxide or ultra-high-density polyethylene, have become invaluable. Within CAD-based reconstructive planning, especially for orbital implants, critical factors include the implant’s anatomical fit, passive stabilization on intact bony structures, and non-interference with orbital soft tissues. Above all, precise replication of the orbital dimensions is essential for optimal clinical outcomes. This study compares the morphological accuracy of orbital structures based on anthropometric measurements from 3D models generated from fan-beam computed tomography (FBCT) and cone-beam computed tomography (CBCT). **Methods:** A cohort group of 500 Caucasian patients aged 8 to 88 years was analyzed. 3D models of the orbits were generated from FBCT and CBCT scans. Anthropometric measurements were taken to evaluate the morphological accuracy of the orbital structures. The assessed parameters included orbital depth, orbital width, the distance from the infraorbital rim to the infraorbital foramen, the distance between the piriform aperture and the infraorbital foramen, and the distance from the zygomatico-orbital foramen to the infraorbital rim. **Results:** Statistically significant differences were observed between virtual models derived from FBCT and those based on CBCT in several key parameters. Discrepancies were particularly evident in measurements of orbital depth, orbital width, the distance from the infraorbital rim to the infraorbital foramen, the distance between the piriform aperture and the infraorbital foramen, and the distance from the zygomatico-orbital foramen to the infraorbital rim. **Conclusions:** The statistically significant discrepancies in selected orbital dimensions—particularly in regions of so-called thin bone—demonstrate that FBCT remains the gold standard in the planning and design of CAD/CAM patient-specific orbital implants. Despite its advantages, including greater accessibility and lower radiation dose, CBCT shows limited reliability in the context of orbital and infraorbital reconstruction planning.

## 1. Introduction

The human orbit is a complex pyramidal bony structure formed by seven craniofacial bones: frontal, sphenoid (both greater and lesser wings), zygomatic, maxillary, ethmoid, lacrimal, and palatine. These elements constitute four anatomically distinct walls: superior (roof), inferior (floor), medial, and lateral. Each wall differs in thickness and mechanical resistance, which are critical in surgical planning and radiological assessment [1].

Orbital reconstruction is a recurring topic across several disciplines within the field of maxillofacial surgery, including trauma surgery, oncologic reconstruction of the mid- and upper facial third, and zygomatic implantology. Each of these clinical contexts poses unique challenges that require precise anatomical understanding and surgical foresight [2].

Three-dimensional (3D) printed models have become an integral component of preoperative planning, providing a tangible representation of complex anatomical structures. In a similar manner, the design of patient-specific implants requires the use of accurate stereolithographic models derived from cross-sectional imaging. These models are typically generated through the segmentation of either fan-beam computed tomography (FBCT) or cone-beam computed tomography (CBCT) scans. In this study, the fan-beam CT (FBCT) group consisted of scans acquired with multi-detector spiral CT (MSCT), which is the standard modality for high-resolution orbital imaging. The segmentation process is pivotal, as it establishes the foundation for surgical templates, intraoperative navigation aids, and custom implant fabrication. In the context of orbital structures, the accuracy of anatomical replication is fundamental. Compared to FBCT, CBCT provides lower soft-tissue contrast and is more susceptible to resolution loss in thin bone regions; however, it remains in use due to its accessibility and lower radiation dose [3].

The bones forming the orbit are perforated by numerous anatomical openings that transmit important neurovascular structures. These include, among others, the anterior and posterior ethmoidal foramina, the optic canal, and the zygomatico-orbital canal. Injury to any of these structures can lead to various functional complications [4].

For instance, damage to the ethmoidal arteries during surgery involving the medial orbital wall can result in the formation of a retrobulbar hematoma [5]. If not recognized and treated in time, this may lead to orbital compartment syndrome and permanent loss of vision. This risk is particularly important in procedures performed under controlled hypotension, which may give the surgeon a false sense of hemostatic control. Only after blood pressure is normalized can hidden bleeding become apparent [6].

Injury to the zygomatico-orbital nerve during dissection of the lateral wall may cause unpleasant sensory disturbances, such as numbness in the cheek and lateral orbital region—often difficult to resolve postoperatively [7,8].

Special attention should also be given to the infraorbital nerve, which is often affected in trauma and surgical procedures involving the midface. Sensory impairment of this nerve is a common complication of orbital fractures, Le Fort I osteotomies, midface trauma surgeries performed through intraoral access, midfacial degloving, and placement of zygomatic or nasal implants [9,10,11,12]. Careful preoperative evaluation of the infraorbital nerve’s course and condition is essential to reduce the risk of this type of complication.

The aim of this study was to compare the accuracy of 3D orbital models derived from CBCT and FBCT scans in order to evaluate their reliability for use in surgical planning and implant design.

## 2. Materials and Methods

This study was conducted with the approval of the institutional bioethics committee (approval numbers: NN/266/11/KB, RNN/267/11/KB, and RNN/141/12/KB).

A total of 499 orbits from individuals of Caucasian descent, aged between 8 and 88 years, were included in the analysis. The imaging data were acquired using two types of scanners: a cone-beam computed tomography (CBCT) system (Carestream CS 9300 3D, Carestream Dental LLC, Atlanta, GA, USA) and a fan-beam multidetector CT (MDCT) system (Aquilion ONE, Toshiba, Otawara, Japan). The dataset consisted of 66 CBCT and 184 MDCT scans, all of which were anonymized prior to analysis, in accordance with established protocols [13].

Cases were selected from the database of the Maxillofacial Surgery Clinic. Exclusion criteria involved congenital orbital anomalies (such as sphenoid wing dysplasia or Goldenhar syndrome), pathological bone remodeling (e.g., tumor-related changes), and any history of trauma, orbital surgery (including reconstructive procedures or implant placement), or partial orbital resection. Scans with poor image quality or significant artifacts were also excluded. Twelve scans were excluded due to segmentation errors or poor quality, resulting in a final sample of 499 orbits.

**Figure 1:**
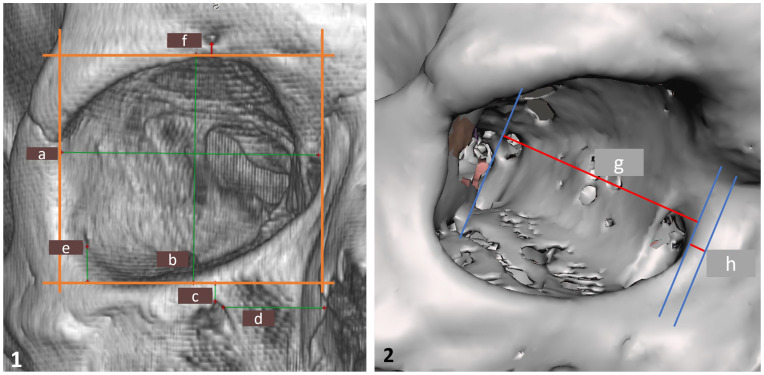
(**1**). Three-dimensional model generated in RadiAnt 2024.2 software, shown in frontal projection. Visible measurements: a—orbital width; b—orbital height; c—distance from the infraorbital rim to the infraorbital foramen; d—distance from the piriform aperture to the infraorbital foramen; e—distance from the zygomatico-orbital foramen to the infraorbital rim; and f—distance from the supraorbital foramen to the supraorbital rim. (**2**). Three-dimensional model with measurements performed in Meshmixer software v.3.5.474 shown in oblique lateral (¾) projection: h—orbital depth; g—bone thickness anterior to the nasolacrimal duct.

DICOM axial image sets were processed to generate three-dimensional bone models through segmentation. Bone structures were extracted using global thresholding values determined separately for CBCT and FBCT datasets, based on individual histogram assessments as per the method of Baillard and Barillot [14]. The resulting 3D models were then used for linear measurements. All steps—from segmentation to measurement—were carried out using RadiAnt DICOM Viewer (Medixant, Poznań, Poland; www.radiantviewer.com, accessed 9 April 2024) and Meshmixer (Autodesk, San Rafael, CA, USA; www.autodesk.com, accessed 9 April 2024) (Figure 1).

For measurement purposes, all models were first aligned along the Frankfurt horizontal plane and then adjusted to a 0-degree frontal projection. The orbital walls were delineated as perpendicular planes, and linear measurements (a–f) were taken in RadiAnt software 2024.2, oriented parallel to the respective anatomical boundaries.

A similar approach was used in Meshmixer v.3.5.474, where the models were rotated in the axial plane to obtain a ¾ lateral view. A tangent line was drawn along the impression of the lacrimal sac on the medial orbital wall, followed by two parallel reference lines: one passing through the anterior margin of the medial orbital rim, and the other positioned posterior to the optic canal. Measurements g and h were taken perpendicularly between these reference lines.

In this study, orbital width and orbital depth were designated as primary variables due to their relevance in clinical planning and implant fit. Secondary variables included distances related to neurovascular foramina and measurements of bone thickness.

Statistical analysis was conducted using Statgraphics Centurion XVIII software. Relationships between categorical variables were evaluated using the chi-squared (χ^2^) test. For continuous variables, one-way ANOVA was applied when assumptions of normality and homogeneity of variance were met; otherwise, the Kruskal–Wallis test was used. A *p*-value of less than 0.05 was considered statistically significant.

## 3. Results

A total of 499 orbital models were included in the study, comprising 363 models derived from fan-beam CT (FBCT) and 124 models obtained from cone-beam CT (CBCT). The CBCT group consisted of 72 female and 60 male patients, while the FBCT group included 110 women and 257 men. Analysis with the chi-square test revealed a statistically significant predominance of male patients across the dataset (*p* < 0.05).

**Figure 2:**
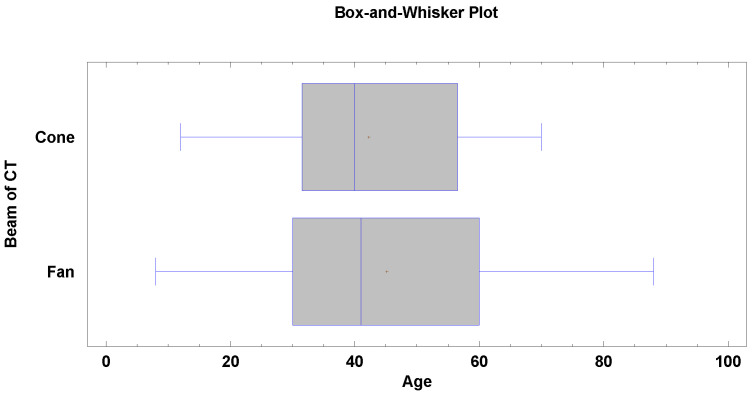
Results indicating statistically insignificant (*p* > 0.05) differences between the age of patients and the type of CT subjected to segmentation.

No significant age difference was observed between the CBCT and FBCT groups (*p* > 0.05). The median age in the CBCT cohort was 40 years (±14.5), while in the FBCT group it was 41 years (±18.9), as indicated in Figure 2.

Out of the initial 499 CT scans, 12 were excluded from the measurement phase due to insufficient image quality or segmentation issues.

As shown in Table 1, the Kruskal–Wallis test revealed no statistically significant differences between CBCT- and FBCT-based models in terms of orbital height, bone thickness anterior to the nasolacrimal duct, and the distance from the supraorbital foramen to the supraorbital rim (*p* > 0.05).

However, statistically significant differences (*p* < 0.05) were found in the measurements of orbital width, the distance from the infraorbital rim to the infraorbital foramen, and the distance from the zygomatico-orbital foramen to the infraorbital rim, depending on the type of imaging used.

**Figure 3:**
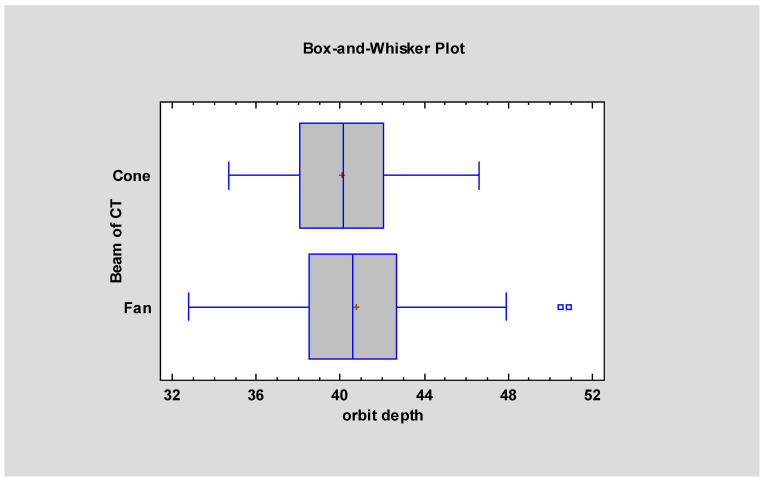

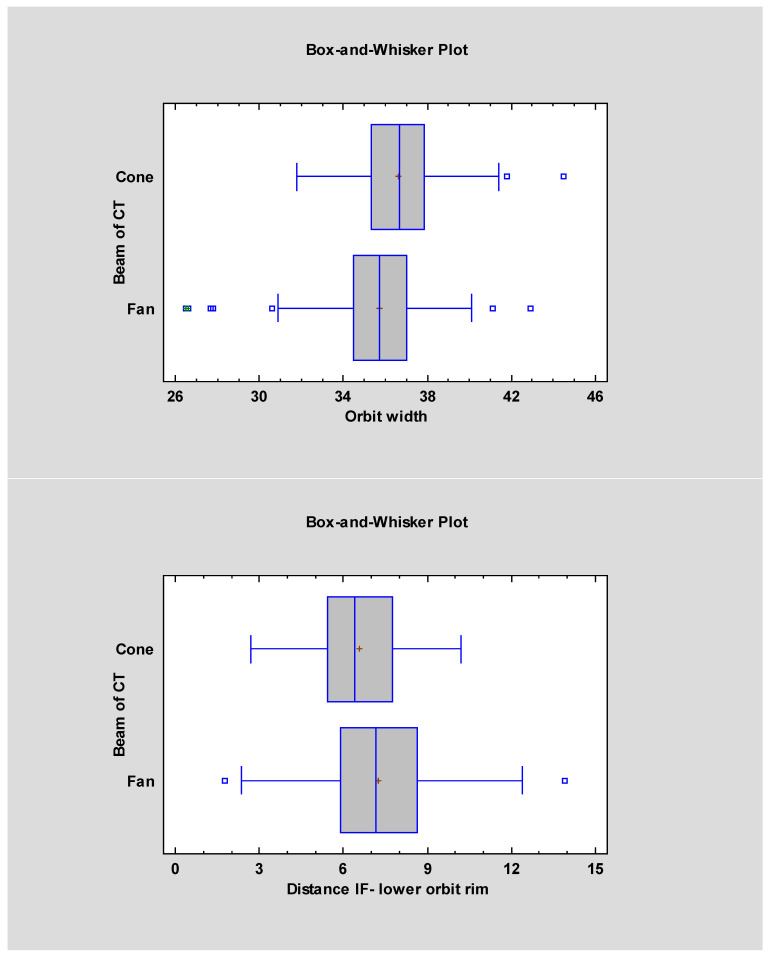

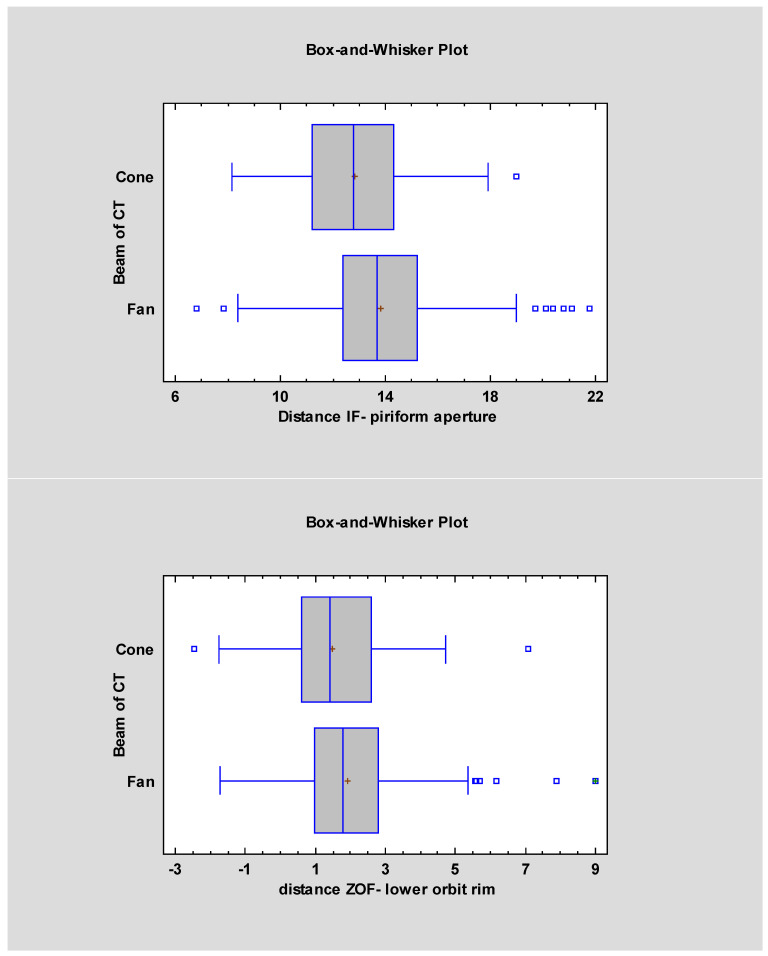
Measurements showing statistically significant differences (*p* < 0.05) between models segmented from cone-beam and fan-beam CT data.

Analysis of variance (ANOVA) revealed statistically significant differences between CBCT- and FBCT-based models in two parameters: orbital depth and the distance between the piriform aperture and the infraorbital foramen (*p* < 0.05). These findings are illustrated in Figure 3.

## 4. Discussion

The use of patient-specific implants in orbital reconstruction—whether in trauma cases, pathology-related defects, or post-oncologic reconstructions—has been shown to reduce hospitalization time and improve functional outcomes. This is true both for customized implants manufactured via CAD/CAM technology and for manually contoured titanium meshes shaped on stereolithographic models [15].

A critical factor in achieving optimal reconstruction outcomes is the geometric accuracy of the model relative to the patient’s actual bone anatomy [16,17,18]. Several variables influence this accuracy, including the resolution of the imaging device [19], voxel size [20], and the type of CT technology used for acquisition [21].

Fan-beam computed tomography (FBCT) remains the gold standard for imaging the craniofacial skeleton, as it provides highly reliable dimensional fidelity [22]. In contrast, models generated from cone-beam CT (CBCT) tend to show reduced accuracy in replicating fine anatomical details, particularly in thin bony regions [23]. These models often underestimate true bone dimensions, and this discrepancy becomes more pronounced with the increase in the field of view (FoV) [19]. This is of clinical relevance, as capturing the entire orbit typically requires one of the largest available FoV settings (e.g., 12 × 12 cm, 14 × 10 cm, or 16 × 13 cm).

Despite clear advantages of CBCT—including greater availability, lower radiation dose, and reduced susceptibility to metal artifacts—it remains essential for surgeons to interpret CBCT-based orbital models with caution, particularly when working in areas of thin bone [21]. This is especially relevant when planning procedures that rely on precise fit and contouring of implants. Table 2 highlights the regions where reduced bone thickness may introduce the highest risk of dimensional inaccuracy in CBCT-based models.

Surgeons relying on CBCT alone for preoperative planning may unintentionally misjudge the position of critical structures, such as the infraorbital foramen, which may appear more medially or superiorly located than in reality. This positional shift can lead to an increased risk of infraorbital nerve injury, inadequate implant seating, or compromised fit of patient-specific implants. Special caution is warranted in high-precision applications such as orbital floor reconstruction and zygomatic implant placement, particularly in quad-zygoma configurations.

Whenever possible, FBCT should be used as the primary imaging modality for CAD/CAM-based orbital reconstruction to minimize the risk of intraoperative complications and improve implant fit and function.

In clinical practice, when FBCT is not available and planning must rely on CBCT data, surgeons should consider incorporating expected positional deviations of key anatomical landmarks into the preoperative workflow. Based on our findings, the infraorbital foramen and other critical structures may appear 0.5–1.0 mm closer to the orbital rim and piriform aperture. Recognizing and adjusting for these systematic shifts may help mitigate intraoperative risk and improve implant fit, even when using CBCT-based models.

In the present study, statistically significant differences were observed in the measurements of orbital width and depth. Notably, both of these dimensions involve anatomical regions where at least one measurement point is located within areas of thin orbital bone, which may influence accuracy in CBCT-derived models.

Although the difference in bone thickness prior to the nasolacrimal duct did not reach statistical significance (*p* = 0.07), a consistent trend was noted in the data, suggesting that CBCT tends to underestimate measurements in this region.

In contrast, no significant differences were found in the dimensions measured along the superior orbital rim or in overall orbital height between models generated from CBCT and those based on FBCT segmentation.

A separate consideration should be given to the anatomical position of the infraorbital foramen, as it transmits the infraorbital nerve—a key sensory branch of the maxillary division of the trigeminal nerve. This structure is of particular clinical importance, as it is implicated in up to 60–75% of postoperative sensory complications following midfacial procedures such as trauma surgery and Le Fort I osteotomies [9,11,24]. Notably, in cases of zygomaticomaxillary complex fractures, infraorbital nerve dysfunction has been reported in 37% to 75% of patients, depending on fracture complexity and surgical approach [9,12]. Moreover, sensory disturbances can persist for up to six months or longer in a significant proportion of cases [24].

The infraorbital foramen is also a crucial surgical landmark during the placement of zygomatic implants. Given the risk of nerve injury and the frequency of sensory impairment in this region, precise localization of the foramen is essential for both planning and execution of surgical procedures involving the midface [25].

When surgical planning relies solely on CBCT-based models, the infraorbital foramen is typically visualized closer to both the orbital rim and the piriform aperture. However, our findings indicate that in models derived from FBCT segmentation, the infraorbital foramen is located lower and more distally relative to the piriform aperture. Given that FBCT is generally considered more accurate in replicating true anatomical structures, this discrepancy suggests that surgeons working with CBCT data may encounter the infraorbital nerve earlier—i.e., in a more medial and superior position—during dissection toward the inferior orbital rim.

This positional shift may also influence implant trajectory, particularly in complex cases involving quad-zygoma configurations, where the more lateral placement of the foramen (as seen in FBCT) may conflict with the planned implant entry point.

A similar discrepancy was observed with the zygomatico-orbital foramen. This relationship resembles a mirror image, with the inferior orbital rim acting as the axis of reflection: just as the infraorbital foramen is found closer to the rim in CBCT-based models, the zygomatico-orbital foramen is also positioned lower—i.e., closer to the rim—than in FBCT-derived models, where it is located higher on the lateral orbital wall.

Further studies are warranted to assess the clinical impact of dimensional discrepancies between CBCT- and FBCT-based planning in live surgical outcomes. Comparative trials correlating intraoperative navigation data and postoperative results with the type of imaging used would help define clearer guidelines for image modality selection in orbital surgery.

One of the limitations of our study is the unequal male-to-female ratio in both imaging groups, which may affect the comparability of orbital dimensions due to known sex-based anatomical variability [26,27].

## 5. Conclusions

This study demonstrated that fan-beam CT (FBCT) provides significantly greater accuracy than cone-beam CT (CBCT) in replicating key orbital dimensions, particularly in regions composed of thin or fragile bone. Measurements such as orbital width, depth, and distances involving the infraorbital and zygomatico-orbital foramina showed consistent underestimation in CBCT-derived models. These discrepancies, though sometimes subtle, are clinically relevant in procedures requiring millimetric precision.

While CBCT remains a valuable diagnostic tool—particularly due to its accessibility, lower radiation dose, and metal artifact reduction—its limitations in orbital imaging should be acknowledged in contexts where exact anatomical reproduction is essential.

In addition, advances in CBCT segmentation algorithms and voxel resolution may, in the future, narrow the accuracy gap between these two modalities—offering a safer and more accessible alternative for high-resolution anatomical modeling.

## Figures and Tables

**Table 1:** Obtained results of measurements of the orbit region in the two radiological imaging techniques.

Measurement Names	FBCT Mean ± SD	FBCT Median	CBCT Mean ± SD	CBCT Median	Statistical Significancy
Orbital width	36.61 ± 2.01	35.7	35.74 ± 2.05	36.7	*p* < 0.05
Orbital height	33.65 ± 2.23	33.4	34.02 ± 2.17	34.05	
Orbital depth	40.75 ± 3.05	40.75	40.07 ± 2.66	40.15	*p* < 0.05
Bone thickness prior to nasolacrimal duct	3.69 ± 1.07	3.5	3.45 ± 1.01	3.38	
The distance from the infraorbital rim to the infraorbital foramen	7.26 ± 1.99	7.17	6.57 ± 1.7	6.42	*p* < 0.05
The distance from the piriform aperture and the infraorbital foramen	13.82 ± 2.36	13.7	12.82 ± 2.21	12.8	*p* < 0.05
Distance from the zygomatico-orbital foramen to the infraorbital rim	1.93 ± 1.53	1.8	1.49 ± 1.56	1.43	*p* < 0.05
Distance from supraorbital foramen to the supraorbital rim	2.01 ± 1.43	1.43	1.54 ± 1.52	1.52	

**Table 2:** Comparison of orbital bone thickness with clinical considerations.

Bone	Orbital Location	Thin Bone?	Clinical Remarks
Ethmoid bone (lamina papyracea)	Medial wall	✅ Yes	Thinnest structure (<0.2 mm)
Maxillary bone (orbital floor)	Inferior wall	✅ Yes	Particularly thin medially
Lacrimal bone	Anterior part of medial wall	✅ Yes	Small and fragile
Palatine bone (orbital process)	Posteromedial part of orbital floor	✅ Yes	Thin and difficult to visualize
Frontal bone	Superior wall	❌ No	Thick and structurally stable
Zygomatic bone	Lateral wall and inferior orbital rim	❌ No	Robust, supports patient-specific implants (PSI)
Sphenoid bone (wings)	Superior and lateral walls	❌ No	Dense, central bone of the skull base

## Data Availability

The original contributions presented in this study are included in the article. Further inquiries can be directed to the corresponding author.
